# Class-3 Semaphorins and Their Receptors: Potent Multifunctional Modulators of Tumor Progression

**DOI:** 10.3390/ijms20030556

**Published:** 2019-01-28

**Authors:** Shira Toledano, Inbal Nir-Zvi, Rotem Engelman, Ofra Kessler, Gera Neufeld

**Affiliations:** The Technion Integrated Cancer Center, The Bruce Rappaport Faculty of Medicine, Technion-Israel Institute of Technology, Haifa 31096, Israel; shiratoledano1@gmail.com (S.T.); inbalnz37@gmail.com (I.N.-Z.); rotemv6@gmail.com (R.E.); ofrak4@gmail.com (O.K.)

**Keywords:** semaphorins, angiogenesis, cancer, lymphangiogenesis, neuropilins, plexins

## Abstract

Semaphorins are the products of a large gene family containing 28 genes of which 21 are found in vertebrates. Class-3 semaphorins constitute a subfamily of seven vertebrate semaphorins which differ from the other vertebrate semaphorins in that they are the only secreted semaphorins and are distinguished from other semaphorins by the presence of a basic domain at their C termini. Class-3 semaphorins were initially characterized as axon guidance factors, but have subsequently been found to regulate immune responses, angiogenesis, lymphangiogenesis, and a variety of additional physiological and developmental functions. Most class-3 semaphorins transduce their signals by binding to receptors belonging to the neuropilin family which subsequently associate with receptors of the plexin family to form functional class-3 semaphorin receptors. Recent evidence suggests that class-3 semaphorins also fulfill important regulatory roles in multiple forms of cancer. Several class-3 semaphorins function as endogenous inhibitors of tumor angiogenesis. Others were found to inhibit tumor metastasis by inhibition of tumor lymphangiogenesis, by direct effects on the behavior of tumor cells, or by modulation of immune responses. Notably, some semaphorins such as sema3C and sema3E have also been found to potentiate tumor progression using various mechanisms. This review focuses on the roles of the different class-3 semaphorins in tumor progression.

## 1. The Class-3 Semaphorin Subfamily

**Figure 1:**
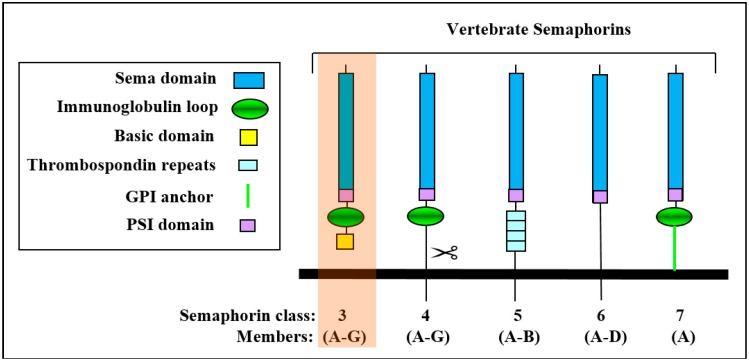
The vertebrate semaphorins: Shown are the main structural features of the sub-families of the vertebrate semaphorins. The 21 members of the vertebrate semaphorin family all contain the hallmark sema domain and are divided into five subfamilies based upon structural features. The seven class-3 semaphorins are the only secreted semaphorins and are also distinguished from the other semaphorins by their basic c-terminal domain.

Class-3 semaphorins (sema3A-3G) are characterized, like all semaphorins, by the presence of a ~500 amino-acid-long sema domain located close to their N-termini which is also present in semaphorin receptors of the plexin family. Like all semaphorins, they also contain a plexin-semaphorin-integrin (PSI) domain located downstream to the sema domain. They also contain an immunoglobulin-like domain and are distinguished from other semaphorins by the presence of a basic domain located downstream to the PSI domain (Figure 1). Class-3 semaphorins are the only vertebrate semaphorins that are produced as secreted proteins while other vertebrate semaphorins are membrane anchored or trans-membrane proteins that can sometimes be further processed into soluble forms by proteolytic cleavage (Figure 1) [1]. The sema domain is essential for semaphorin activity and plays a role in the determination of the receptor binding specificity [2]. The sema domains of several different semaphorins have been characterized by X-ray crystallography revealing a beta propeller topology [3,4,5]. The active forms of the class-3 semaphorins are homo-dimeric [6,7,8,9]. All class-3 semaphorins contain at least two conserved basic cleavage sites for furin-like pro-protein convertases (FPPC). A major cleavage site is located downstream to the sema domain and another cleavage site is located in the basic domain [10]. Cleavage of different class-3 semaphorins at the major cleavage site usually inactivates them [10,11]. However, there are exceptions as in the case of sema3E in which the cleaved product retains full activity [12,13,14]. In contrast, cleavage in the basic domain potentiated the activity of sema3A [10] and there are reports suggesting that cleavage at this site may be essential for the anti-angiogenic activities of sema3F and sema3C [15,16].

## 2. Class-3 Semaphorin Receptors

### 2.1. The Neuropilins: Multifunctional Scaffold Receptors For Class-3 Semaphorins

**Figure 2:**
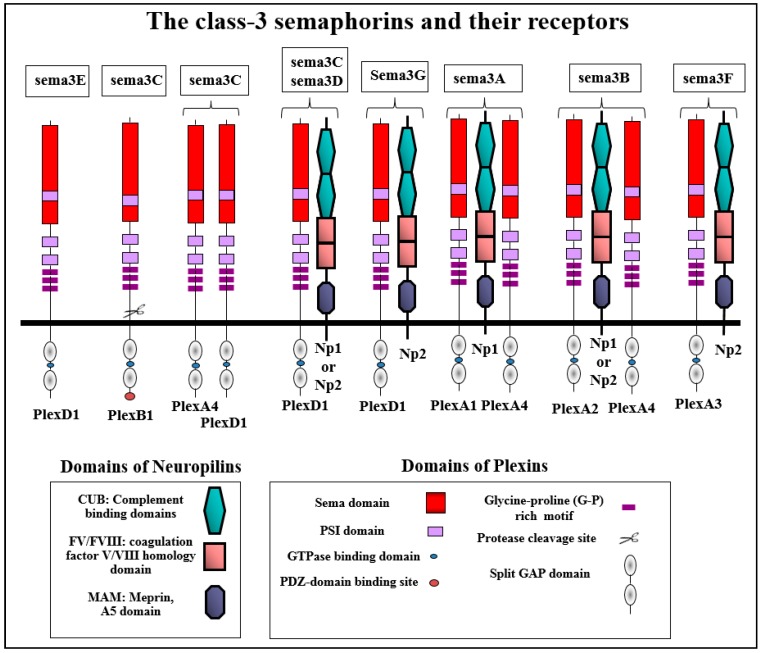
The class-3 semaphorins and their receptors: The compositions of the functional receptor complexes that transduce the signals of the different class-3 semaphorins to induce cytoskeletal collapse in different cell types are shown. Plex stands as an abbreviation for plexin and Np as an abbreviation for neuropilins. Shown also are the main structural elements of the different plexins and neuropilins that function as class-3 semaphorin receptors. Neuropilins and plexins are single-pass transmembrane receptors. Neuropilins contain two complement-like binding domains (CUB domains also known as the a1 and a2 domains) that bind class 3 semaphorins. They contain two FV/FVIII coagulation factor homology domains (also termed the b1 and b2 domains). The b1 domain also participates in the binding of class-3 semaphorins. In contrast, VEGF binds to both. The MAM domain is believed to mediate neuropilin dimerization. The intracellular domain of the neuropilins contains a C-terminal SEA sequence that interacts with the PDZ domain containing proteins such as synectin. Plexins also contain a sema domain in their extracellular domain. They contain PSI (Plexin, Semaphorin, Integrin) motifs and IPT/(G-P)-rich motifs involved in the binding of semaphorins. The intracellular part contains a split GTPase activating (GAP) domain separated by a GTPase-binding domain that binds small GTPases such as Rac.

Most of the vertebrate semaphorins bind to one of the nine receptors of the plexin family which function as the main transducers of their signals [17]. However, most of the class-3 semaphorins, with the exception of sema3E [18], utilize as their main binding receptor one or both receptors of the neuropilin family [1,19,20,21]. Sema3A binds to neuropilin-1 exclusively [19,22]. Sema3F and sema3G signal using neuropilin-2 [23] while sema3B, sema3C and sema3D bind to both neuropilins (Figure 2) [23]. However, binding to neuropilins is not sufficient to transduce class-3 semaphorin signals due to the short intracellular domains of the neuropilins. To transduce class-3 semaphorin signals, the neuropilins form complexes with one or more of the four type-A plexins or with plexin-D1 [17,21,24]. In these functional class-3 semaphorin receptor complexes, the plexins serve as the signal transducing components.

The neuropilins can perhaps be best classified as “scaffold receptors” since they are able to bind with high affinity to many diverse ligands including several forms of vascular endothelial growth factor-A (VEGF-A) [25,26,27], VEGF-B, VEGF-C [28,29], placental growth factor (PLGF) [30], hepatocyte growth factor (HGF) [31], platelet derived growth factor PDGF-D [32], transforming growth factor-β (TGF-β) [33] as well as galectin-1 [34] in addition to several class-3 semaphorins. In addition, the neuropilins can form complexes with many types of membrane-bound receptors in addition to various plexins and can modulate signal transduction by these receptors. These include the VEGF tyrosine-kinase receptors VEGFR-1, VEGFR-2 and VEGFR-3 [26,29,35,36], The platelet derived growth factor tyrosine-kinase receptors PDGFR-β and PDGFR-α [37,38], Neurotrophin receptors [39], integrins 1β and αVβ3 [40,41], The cell adhesion molecule (CAM) L1-CAM, close homolog of L1-CAM, and NrCAM [42,43,44], the hepatocyte growth factor receptor MET [45], and TGF-β receptors [33].

The VEGF-A binding domain of neuropilin-1 seems to be distinct from its class-3 semaphorin binding domain [46]. Indeed, sema3A inhibits VEGF-induced activation of the extracellular signal–regulated kinase ERK1/2 but does not inhibit VEGF-induced auto-phosphorylation of the VEGFR2 receptor suggesting independently that sema3A does not compete with VEGF-A for binding to neuropilin-1 [47]. However, there is also evidence suggesting that some class-3 semaphorins may compete with VEGF family members for binding to neuropilins [48] and that post-translational modifications of semaphorins such as cleavage by furin-like pro-protein convertases [10] may modulate their neuropilin binding ability and their ability to complete with VEGFs for binding to neuropilins [49]. Recent experiments reveal that the short intracellular domain of neuropilin-1 is nevertheless essential for some functions since mice in which the neuropilin-1 gene was replaced with neuropilin-1 lacking the intracellular domain have a defect in arteriogenesis [50]. The intracellular domain of neuropilin-1 was also found to be important for the interaction of myofibroblasts with soluble fibronectin, an interaction that promotes alpha5/beta1 integrin-dependent fibronectin fibril assembly [51]. The intracellular domain of neuropilin-1 contains a PDZ binding domain which binds synectin (also known as GIPC or NIP) and this interaction is important for the formation of complexes with VEGFR-2 [52,53].

### 2.2. Plexin Receptors: Signal Transducers of Class-3 Semaphorins

The nine receptors of the plexin family are single pass receptors that are segregated into four groups consisting of four Type-A plexins, three Type-B plexins, and single C and D plexins [54]. The four type-A plexins and plexin-D1 form complexes with neuropilins and have been characterized as components of functional class-3 semaphorin receptors (Figure 2). The extracellular domains of all plexins contain a sema domain which serves as an auto-inhibitory domain in the basal, non-activated state of the receptor [55]. The intracellular parts of all plexins are characterized by the presence of a GTPase activating protein (GAP) domain which is conserved throughout the plexin family [56,57,58]. They also contain in their intracellular domain a split cytoplasmic SP (sex-plexin) domain as well as putative tyrosine phosphorylation sites. However, they do not possess a tyrosine kinase domain. In the cases of plexin-D1 and plexin-B1, it was demonstrated that most of the developmental effects of these plexins are lost if the function of the GAP domain is compromised [59]. Type-A plexins associate spontaneously to form homodimers [9,60]. Recent data indicates that the activation of plexin signaling by semaphorins that bind directly to plexins such as sema6A is associated with a change in the spatial organization of the plexin dimers, shifting the conformation from the inactive to the active form [5,9]. In the case of class-3 semaphorin sema3A, there is structural evidence suggesting that when plexin-A2 and neuropilin-1 are over-expressed they form a functional tetrameric sema3A receptor composed of two plexin-A2 receptors and two neuropilin-1 receptors following stimulation with sema3A [7]. However, functional sema3A receptors that are found in living cells probably contain more than one type-A plexin in addition to a neuropilin. Thus, sema3A-induced cytoskeletal collapse in endothelial cells is completely inhibited following the silencing of either neuropilin-1, plexin-A1 or plexin-A4 but not by the silencing of other type A plexins (Figure 2) [61]. These observations are supported by studies in which it was observed that sema3A signaling is impaired in mice lacking functional plexin-A4 or plexin-A1 receptors [62,63,64,65]. Likewise, cytoskeletal collapse induced by sema3B is completely abrogated if the expression of both neuropilins or plexin-A2 or plexin-A4 is inhibited [66]. These observations suggest that the functional receptor complex transducing sema3A-induced cytoskeletal collapse consists of a complex containing plexin-A4, plexin-A1 and neuropilin-1, all of which are required for signal transduction, while the receptor complex of sema3B consists of a complex containing a neuropilin, plexin-A4 and plexin-A2 (Figure 2). However, at higher levels of expression, some plexins can replace other plexins. For example, plexin-A2 can replace plexin-A1 in sema3A receptors when it is highly expressed in cells indicating that there is a significant level of plasticity that enables signal transduction under diverse conditions [66]. Notably, different compositions of functional receptors may be assembled for the transduction of different signals by a given semaphorin. For example, it was observed that plexin-A3 rather than plexin-A4 is required for the transduction of sema3A signals triggering neuronal apoptosis [67].

Based upon their interaction with neuropilins and plexins, class-3 semaphorins can be divided into several sub-categories. Sema3E differs from the other class-3 semaphorins as it is the only class-3 semaphorin that does not bind to neuropilins and binds directly to plexin-D1 to transduce its signals [18]. Three additional class-3 semaphorins seem to transduce their signals primarily through plexin-D1. These are sema3C, sema3D and sema3G (Figure 2) [23,68,69]. Sema3D and sema3G also require in addition to plexin-D1 a neuropilin in order to transduce their signals [23]. However, there are also experiments suggesting that sema3D may also be able to transduce signals using additional plexins [70]. Notably, sema3C differs from sema3G and sema3D in that it is also able to transduce signals in the absence of neuropilins using functional receptors containing plexin-A4 and plexin-D1, although under these conditions a five-fold higher concentration of sema3C is required for signal transduction [23]. Furthermore, it was recently observed that unlike other class-3 semaphorins, sema3C can also bind to and transduce signals using the plexin-B1 receptor [71].

Activation of plexin signaling by semaphorins results in the activation of the GAP domain of the plexins. This in turn inactivates R-ras, resulting in the subsequent inactivation of beta1-integrin and dissociation from the extracellular matrix [58,72,73,74]. The activation of type-A plexins also results in the activation of enzymes of the Mical family which oxidize actin subunits leading to the disassembly of actin fibers and to localized collapse of the actin cytoskeleton of axonal growth cones [75,76,77,78] as well as the activation of various intracellular tyrosine-kinases [79] and the inactivation of the small GTPase RhoA that subsequently leads to the activation of cofilin and the de-polymeriztion of the actin cytoskeleton [1,59,80].

## 3. Class-3 Semaphorins as Regulators of Tumor Progression

### 3.1. Modulation of Tumor Angiogenesis by Class-3 Semaphorins

The first hint that suggested that class-3 semaphorins may perhaps function as modulators of angiogenesis came when it was observed that neuropilin-1 and neuropilin-2 also function as receptors for the angiogenesis promoting factor vascular endothelial growth factor-A (VEGF-A) [26,27]. The progression of solid tumors depends upon angiogenesis [81], and it was indeed initially found that sema3F, a semaphorin that utilizes the neuropilin-2 receptor as its primary binding receptor [20], functions as an inhibitor of angiogenesis and of angiogenesis-dependent tumor progression [82,83]. It was subsequently found that additional class-3 semaphorins such as sema3A [84], sema3B [11], sema3C [48,85], sema3D [86], and sema3E [14,58] also function as potent anti-angiogenic factors. Furthermore, some of the class-3 semaphorins such as sema3A were characterized as natural inhibitors of angiogenesis whose down-regulation can enable the angiogenic switch that signals the onset of angiogenesis-dependent tumor progression [87,88]. Indeed, it was recently reported that a mutated sema3A, that unlike natural sema3A binds directly to the plexin-A4 receptor and not to neuropilin-1, is a better inhibitor of angiogenesis as compared to wild type sema3A and strongly inhibits the progression of pancreatic cancer in mouse models [89]. Interestingly, it was recently found that sema3A may also function as an inhibitor of the development of hematological malignancies such as multiple myeloma and various forms of leukemia and that this activity is likely also due to its anti-angiogenic activity [90,91,92].

**Figure 3:**
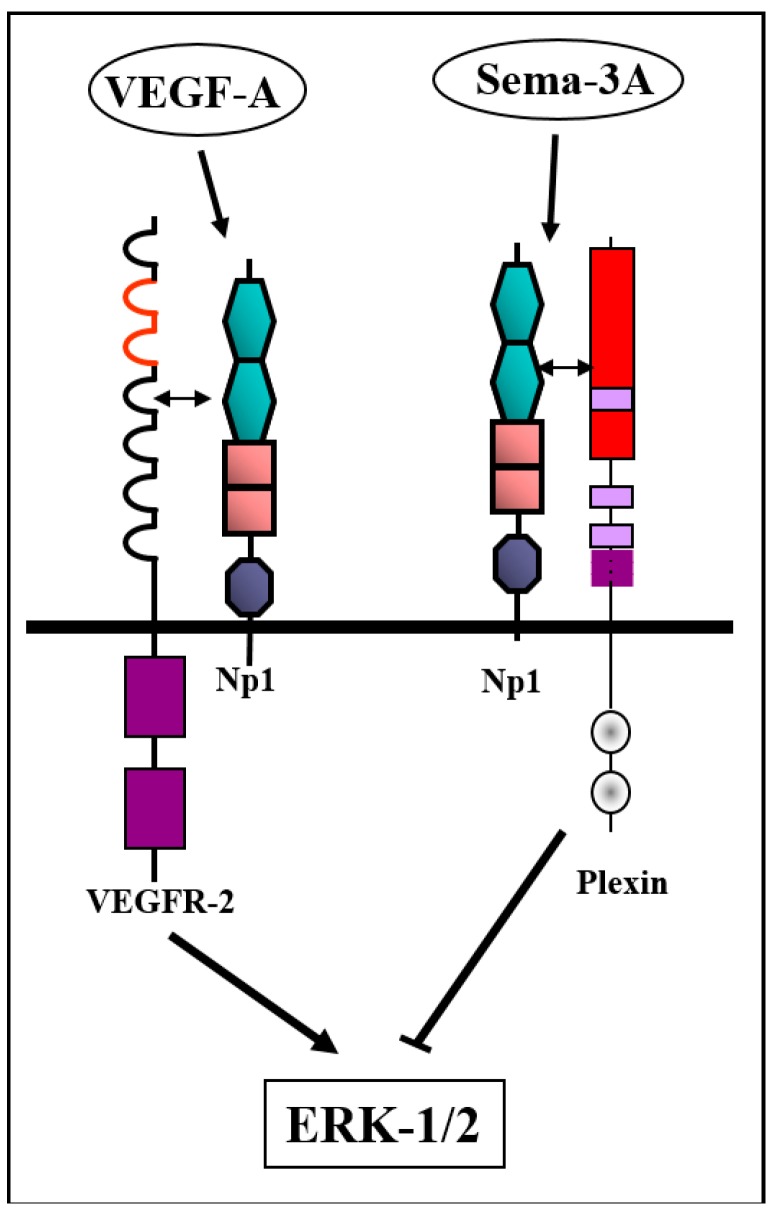
Sema3A inhibits VEGFR-2-mediated pro-angiogenic signaling by activation of an inhibitory intracellular signaling cascade: Sema3A binds to its neuropilin-1 receptor, thereby activating type-A plexins. This inhibits VEGF-A-induced phosphorylation of extracellular signal–regulated kinase-1/2 (ERK-1/2). However, sema3A does not compete with VEGF-A for binding to neuropilin-1 since the binding sites are completely separate. Indeed, sema3A does not inhibit VEGF-A-induced auto-phosphorylation of VEGFR-2 suggesting independently that the inhibition does not take place at the level of the VEGF-A receptors.

The mechanism by which class-3 semaphorins inhibit angiogenesis is not completely resolved and may not be the same for all class-3 semaphorins. In the case of sema3A, it seems that the inhibition is not due to competition with factors such as VEGF-A for binding to neuropilin-1 but is due to the activation of intracellular signaling cascades that inhibit the propagation of signaling cascades activated by pro-angiogenic factors such as VEGF-A (Figure 3) [47]. Similarly, sema3E which does not bind to neuropilins or tyrosine-kinase coupled VEGF receptors [18] nevertheless inhibits VEGF-A-induced activation of ERK [93] and VEGF-A-induced angiogenesis [94,95]. Finally, structural studies indicate that VEGF-A and sema3A bind to non-overlapping sites on neuropilin-1, arguing against a competition-based anti-angiogenic mechanism [46,96]. However, there are also indications that some class-3 semaphorins do compete with pro-angiogenic factors for binding to neuropilins. Thus, it was reported that cleavage of sema3F at its C-terminal by furin-like pro-protein convertases enables its binding to neuropilin-1 and competition with VEGF-A for binding to neuropilin-1 [15,49].

Class-3 semaphorins repulse target cells by induction of a localized collapse of the actin cytoskeleton and by the disruption of local focal adhesions to extracellular matrix ECM components. In vivo, class-3 semaphorins can repulse endothelial cells and consequently inhibit the growth of new blood vessels into areas in which they are expressed. Thus, the expression of sema3E in somites of developing embryos repels blood vessels and keeps the somites avascular [18]. Likewise, repulsion of endothelial cells by sema3E is critical for the formation of the dorsal aorta during embryonic development [97]. Thus, expression of class-3 semaphorins by tumor cells may repulse endothelial cells in the tips of vascular angiogenic sprouts and thus, inhibit tumor angiogenesis and tumor growth. It follows that class-3 semaphorins may function as secreted tumor suppressors. Indeed, sema3F and sema3B have been characterized as bona fide tumor suppressors of lung cancer [98,99,100,101], and sema3A has been found to function as an endogenous angiogenesis inhibitor that is down-regulated during tumor progression [88]. In the case of sema3F, it was found that its expression is under the control of p53. Loss of p53 activity which is frequently encountered in tumor cells results in down-regulation of sema3F expression, thus alleviating sema3F-induced inhibition of angiogenesis and promoting tumor growth, suggesting that sema3F also functions as an endogenous inhibitor of angiogenesis [102]. In addition, it was observed that stimulation with semaphorins can result in addition to the repulsion of cells, also in apoptosis of target cells such as endothelial cells [47,103,104,105,106]. It is thus possible that part of the anti-angiogenic effect of class-3 semaphorins is due to class-3 semaphorins-induced apoptosis of endothelial cells, although the molecular mechanism by which class-3 semaphorins induce apoptosis is unclear.

Little is known about the roles of sema3D and sema3G in tumor progression. Like other class-3 semaphorins, sema3D seems to inhibit tumor progression [86,107,108] and inhibit angiogenesis [86], although there is also some evidence suggesting that it too can have a dual role and promote tumor progression under certain circumstances [109]. However, there are no additional studies that relate to the possible role of sema3D in tumor progression.

Class-3 semaphorins can also sometimes promote tumor angiogenesis and the function of tumor associated blood vessels by indirect mechanisms. Thus, Bone marrow-derived cells can be recruited to sites of active angiogenesis by factors such as stromal cell-derived factor 1 (SDF-1) which are produced at sites of active angiogenesis, and these cells then promote angiogenesis by the secretion of angiogenic factors such as VEGF-A [110]. Sema3A produced by tumor cells is also able to recruit bone marrow-derived cells to tumors. These recruited bone marrow cells consist of a neuropilin-1 expressing a sub-population of monocytes and contribute to the stabilization and normalization of tumor vessels by promoting coverage of tumor vessels by mural cells. This inhibits vascular leakiness, resulting in smaller but better perfused and less hypoxic tumors [111,112]. Sema3A also induces the phosphorylation of the VEGFR-1 tyrosine-kinase receptor in a subpopulation of macrophages in a neuropilin-1, plexin-A1 and plexin-A4-dependent manner, resulting in the recruitment of these macrophages to the hypoxic areas of tumors. These macrophages in turn secrete angiogenic factors that promote tumor angiogenesis and tumor progression [113]. Sema3B can also indirectly induce opposite effects and potentiate tumor metastasis as well as tumor angiogenesis in many types of tumors as a result of sema3B-induced expression of interleukin-8, which in turn, induces the recruitment of tumor-associated macrophages and metastatic dissemination to lungs [114]. Since interleukin-8 is a well characterized angiogenic factor [115], it is likely that when expressed it can also counteract the direct anti-angiogenic effects of sema3B.

### 3.2. Effects of Class-3 Semaphorins on Lymphangiogenesis

Solid tumor cells metastasize to distant locations to form metastases by the invasion of blood vessels or by the invasion of lymph vessels [116,117]. Since class-3 semaphorins have been found to display anti-angiogenic properties, it was hypothesized that some class-3 semaphorins may also function as inhibitors of lymphangiogenesis and of lymph vessels-mediated metastasis. To induce lymphangiogenesis, the best characterized lymphangiogenic factor, VEGF-C, binds to neuropilin-2 which forms complexes with the VEGFR-3 tyrosine-kinase receptor to transduce VEGF-C lymphangiogenic signals [29]. Thus, it was hypothesized that class-3 semaphorins that bind to neuropilin-2 such as sema3F, sema3C and sema3G may perhaps modulate lymphangiogenesis. Indeed, both sema3F and sema3C have been observed to inhibit tumor lymphangiogenesis as well as tumor metastasis mediated by lymphatics [48,68,83,118,119,120]. In addition, it was observed that sema3G can inhibit dermal lymphangiogenesis [121] but it is not known if sema3G can affect tumor lymphangiogenesis or tumor metastasis.

### 3.3. Modulation of Tumor Progression by Direct Effects on Tumor Cells

Many types of tumor cells express class-3 semaphorin receptors. Consequently, it was also found that class-3 semaphorins can affect tumor progression by a variety of mechanisms and not only by the modulation of tumor angiogenesis. Sema3A inhibits the migration and spreading of MDA-MB-231 breast cancer cells as well as their ability to form colonies in soft agar, and it also inhibits similarly the invasiveness of prostate cancer cells in in-vitro assays [108,122,123]. In breast cancer cells, sema3A was found to regulate the phosphorylation and nuclear translocation of phosphatase and tensin homolog (PTEN) and the activation of the forkhead transcription factor FOXO-3a. Conversely, over-expression of PTEN and FOXO-3a was found to enhance sema3A expression resulting in inhibition of breast cancer cells migration [124]. In addition, high mobility group box-1 (HMGB1) binds to the sema3A genomic locus and inhibits sema3A expression, resulting in increased migration of tumor cells [125]. These observations suggest that sema3A can inhibit tumor progression by directly affecting the behavior of tumor cells. However, there are also reports of an opposite activity of sema3A. Up-regulation of Sema3A expression promoted tumor growth and tumor progression in a hepatocellular carcinoma (HCC) mouse model by enhancement of the expression of CapG, galectin-3, enolase 2 and Epithelial cell adhesion molecule (EpCAM) [126]. Furthermore, in glioblastoma multiforme and in pancreatic cancer, sema3A promotes rather than inhibits metastatic dissemination [127,128].

Sema3B was initially characterized as a tumor suppressor of lung cancer [99,129,130] and was found to inhibit the progression of additional forms of cancer such as breast cancer, endometrial cancer, osteosarcoma and oral cancer because of the direct effects on the tumor cells [131,132,133]. Sema3B inhibits the anchorage-independent growth of lung cancer cells inducing apoptosis by direct inhibition of the tumor cells [99].

Sema3F was also identified as a tumor suppressor of lung cancer [98,134] and, subsequently, was identified as an inhibitor of the progression of additional types of cancer [131,135,136,137,138]. Single nucleotide polymorphisms of sema3F are associated with increased prostate cancer risk and poor prognosis [100]. When the cDNA encoding sema3F was expressed in lung cancer cells, breast cancer cells or colorectal cancer cells it inhibited their anchorage-free proliferation and invasiveness [108,134,139,140]. In H157 lung cancer cells, sema3F inhibited multiple signaling pathways including Protein kinase B (AKT)/Signal transducer and activator of transcription 3 (STAT3) signaling resulting in the loss of activated αvβ3-integrin [141,142]. In addition, Sema3F inhibited -β1-integrin mediated attachment of A375 melanoma cells by a neuropilin-2-mediated mechanism and suppressed the metastatic spread of cells from tumors derived from these cells [83]. In colorectal cancer cells, it was also found to suppress the stemness of the tumor cells [143]. Taken together, these experiments indicate that sema3F can directly affect the behavior of tumor cells that express appropriate sema3F receptors.

In contrast with other class-3 semaphorins which function primarily as inhibitors of tumor progression, sema3C displays both pro- and anti-tumorigenic properties. Sema3C was identified as the product of a gene that confers non-MDR drug resistance in human cancers [144]. It utilizes both neuropilin-1 and neuropilin-2 as its binding receptors and transduces its signals using primarily the plexin-D1 receptor [24,48]. However, it was also able to bind directly to the plexin-D1 and to the plexin-B1 receptors independently of neuropilins [23,71]. Contrary to other class-3 semaphorins, its expression in tumor cells is usually associated with tumor progression rather than with inhibition of tumor progression in several types of tumors [145,146,147,148,149,150,151,152]. It was recently observed that its pro tumorigenic activity is likely mediated by the plexin-B1 receptor [71]; however, there are also reports that implicate the plexin-D1 and plexin-A2 receptors [152]. Like the other class-3 semaphorins, sema3C too is cleaved in conserved sites by furin-like pro-protein convertases as well as by ADAMTS1 [153]. The major cleavage product generated by furin-like pro-protein convertases, which are usually up-regulated in the tumor microenvironment [154], is unable to induce the collapse of the cytoskeleton of target cells but is still able to support the survival of tumor cells in cell culture [48], suggesting that it may perhaps contribute to tumor progression. However, sema3C also functions as a potent inhibitor of angiogenesis and lymphangiogenesis, which is likely mediated by the activation of plexin-D1-dependent signal transduction in endothelial cells and lymphatic endothelial cells [48,85], resulting in the inhibition of tumor progression.

Sema3G was also found to inhibit the invasion and migration of gliobalstoma cells [155] and to inhibit the development of tumors from MDA-MB-435 breast cancer cells in which it was expressed ectopically, suggesting that it too can directly affect the development of tumors from tumor cells that express sema3G receptors [108].

Sema3E is unique among the class-3 semaphorins in that it is the only class-3 semaphorin that does not bind to neuropilins [18]. Ectopic over-expression of sema3E in a variety of tumor cell types inhibits tumor development from such cells [86,108,156]. Unlike other class-3 semaphorins, it was initially characterized as a pro-metastatic semaphorin [157]. However, sema3E was also found to function as an inhibitor of angiogenesis, like other class-3 semaphorins, and thus, should function as an inhibitor of tumor metastasis [48,85]. Cleavage of sema3E by furin-like pro-protein convertases generates a ~61 kDa cleavage product (p61-Sema3E). Unlike similar cleavage products derived from sema3A or sema3B which lack bioactivity or display strongly reduced activity, p61-Sema3E retains the activity of full-length sema3E, but unlike full length sema3E, also able induces the formation of complexes between plexin-D1 and the ErbB2 tyrosine-kinase receptor resulting in the in-trans activation of ErbB2 signal transduction, which in turn promotes tumor metastasis [14]. This probably explains why sema3E was initially described as a pro-metastatic factor. Indeed, a point-mutated sema3E resistant to cleavage by furin-like pro-protein convertases inhibits angiogenesis and tumor progression but is unable to activate ErbB2 and is unable to promote tumor metastasis [13].

### 3.4. Modulation of Tumor Progression by Mechanisms Involving Stromal Cells Other Than Endothelial Cells

Sema3E can induce inflammation that is mediated by macrophages [158]. It is not clear if this activity is mediated by full length sema3E or if it is mediated by p61-Sema3E. However, Inflammation is a major contributor to tumor progression [159], suggesting that sema3E may also be able to promote tumor progression by modulation of the chronic inflammation that is a hallmark of many types of tumors.

### 3.5. Modulation of Tumor Progression by Class-3 Semaphorins that Modulate Immune Responses

An important aspect of tumor progression is the ability of cancer cells to escape detection and clearance by the immune system. Tumor-associated macrophages (TAMs) are normally characterized as M1 TAMs that express CD11c which function as inhibitors of tumor progression. However, in the tumor microenvironment, they frequently change their gene expression profile and behave as M2 macrophages that secrete pro-angiogenic factors such as VEGF, PlGF and sema4D to promote tumor angiogenesis and tumor progression [160,161]. M2 macrophages also suppress anti-tumor immunity by preventing the activation of dendritic cells (DCs), cytotoxic T lymphocytes (CTLs), and natural killer (NK) cells [161].

Interestingly, despite the anti-angiogenic properties of sema3A, some tumor cells express sema3A. T cells express the neuropilin-1 and plexin-A4 sema3A receptors, and sema3A containing conditioned medium from such tumor cells was observed to inhibit the proliferation of these cells and their activation by anti-Cd3 antibodies [162]. In agreement, plexin-A4 as well as neuropilin-1 knock-out mice exhibited hyperproliferative responses to anti-CD3 stimulation and enhanced T-cell activation [163]. However, tumor cells-derived sema3A was also found to restrict the proliferation of pro-tumorigenic M2 macrophages and increase the proliferation of anti-tumorigenic M1 macrophages. Expansion of M1 macrophages in vivo enhanced the recruitment and activation of natural killer (NK) cells and cytotoxic CD8 T cells to tumors, inhibiting their growth [164]. Thus, sema3A seems to modulate the recognition of tumor cells by the immune system. It is not known if additional class-3 semaphorins can also affect the function of the immune system. Sema3F was reported to regulate the migration of human T-cell precursors [165], sema3E was found to play a role in allergic asthma [166], and several class-3 semaphorins were found to promote the migration of dendritic cells [167]. More information regarding the possible roles of class-3 semaphorins as modulators of immune responses to malignant cells is required.

## 4. Modifications of Class-3 Semaphorins and Class-3 Semaphorin Receptor Genes As Modulators of Tumor Progression

Most class-3 semaphorins function as inhibitors of angiogenesis and, therefore, inactivating mutations in their genes is expected to promote tumor progression. The Sema3B and sema3F genes are located at location 3p21.3, a region that is frequently deleted in small and non-small cell lung carcinoma [168,169]. Indeed, both genes exhibit properties of tumor suppressors [129]. Promoter hyper-methyletion of sema3B was associated with loss of heterozygosity (LOH) in both cell lines and primary tumors in lung cancer and hepatocellular carcinoma, and there is a statistically significant correlation between the sema3B methylation status and LOH at 3p21.3 [170,171,172]. Single nucleotide polymorphisms in the genes encoding sema3B and sema3F were also associated with prostate cancer risk and poor prognosis of prostate cancer [100]. Similarly, reduced expression of sema3B is associated with glioma development and prognosis as well as in renal cancer and gallbladder carcinoma [130,173,174]. Inhibition of the expression of sema3B and sema3F due to chromosomal alterations was also found to contribute to the progression of inflammatory myofibroblastic tumors [175]. Interestingly, substitution of a single nucleotide of sema3B that was found primarily in African-Americans and Latino-Americans but not in Caucasians, results in reduced lung cancer risk for an unknown reason [176].

A polymorphism in the sema3A promoter is associated with adverse responses to radiation therapy in cancer patients for an unknown reason [177]. Sema3A was also observed to promote, rather that inhibit, glioblastoma progression through the induction of tumor cells proliferation and macrophages recruitment and treatment with an anti-semaphorin-3A antibody inhibited the progression of glioblastoma tumors in mouse models [178]. A somatic mutation in the plexin-A1 sema3A receptor was linked to enhanced proliferation and invasion in pancreatic cancer cells in response to sema3A [179]. Reduced copy number of the sema3C gene was found to be associated with increased risk of colorectal carcinoma [180]. Finally, in malignant methothelioma patients, it was observed that deletions at 3p21.1 in which the gene encoding sema3G is located as well as in deletions at 3p21.3 containing the sema3B and sema3F genes were correlated with disease progression [181].

## 5. Conclusions

The class-3 semaphorins were initially found to function as axon guidance factors but were recently found to affect a much wider range of biological processes including angiogenesis, lymphangiogenesis and immune surveillance and to be players in the etiology of many diseases including cancer. The class-3 semaphorins were found to function primarily as potent inhibitors of tumor progression. Their inhibitory activity targets tumor cells directly as well as stromal cell types critical for tumor progression such as endothelial cells. However, there are also some class-3 semaphorins such as sema3C and sema3E that display dual activities and can both induce and inhibit tumor progression due to specific characteristics such as the ability to activate the plexin-B1 receptor in the case of sema3C and the activation of ErbB2 by cleaved sema3E. These observations suggest that interactions between class-3 semaphorin receptors and apparently unrelated receptors such as various tyrosine-kinase receptors as well as post-translational modifications of the semaphorins and their receptors can profoundly affect their biological activities. These interactions and modifications can in turn profoundly affect the course of diseases such as cancer, and a better understanding of these interactions and post translational modifications is required if one considers the development of anti-tumorigenic and anti-angiogenic therapeutic agents that target or utilize semaphorin signal transduction cascades. Notably, class-3 semaphorins as well as semaphorins belonging to additional semaphorin subclasses are involved in the etiology of additional diseases besides cancer and, as a result, there are attempts to target semaphorins in order to treat additional diseases [182]. Research aimed at a better understanding the processing of the class-3 semaphorins and their receptors and research aimed at better characterization of the cross-talk between semaphorins and their receptors and other signal transduction pathways are likely to be a focus of research in the near future. In addition to cancer, class-3 semaphorins play regulatory roles in the development and maintenance of the vascular and neuronal networks of organs such as the retina and kidney. It is likely that the study of the role of class-3 semaphorins in the development of vascular diseases such as the changes observed in diabetes will also become a focus of research in the near future.
